# Immune Score Predicts Outcomes of Gastric Cancer Patients Treated with Adjuvant Chemoradiotherapy

**DOI:** 10.1155/2021/9344124

**Published:** 2021-12-27

**Authors:** Wei Zou, Meng-long Zhou, Ling-yi Zhang, Jia-ning Yang, Wang Yang, Ya-qi Wang, Yu-xi Yi, Gui-chao Li, Zhen Zhang

**Affiliations:** ^1^Department of Radiation Oncology, Fudan University Shanghai Cancer Center, Shanghai 200032, China; ^2^Department of Oncology, Shanghai Medical College, Fudan University, Shanghai 200032, China; ^3^Shanghai Key Laboratory of Radiation Oncology, Shanghai 200032, China

## Abstract

**Background:**

Substantial evidence has demonstrated that tumor-infiltrating lymphocytes (TILs) are correlated with patient prognosis. The TIL-based immune score (IS) affects prognosis in various cancers, but its prognostic impact in gastric cancer (GC) patients treated with adjuvant chemoradiotherapy remains unclear.

**Methods:**

A total of 101 GC patients who received chemoradiotherapy after gastrectomy were retrospectively analyzed in this study. Immunohistochemistry staining for CD3+ and CD8+ T-cell counts in both tumor center (CT) and invasive margin (IM) regions was built into the IS. Patients were then divided into three groups based on their differential IS levels. The correlation between IS and clinical parameters was analyzed. The prognostic impact of IS and clinical parameters was evaluated using Kaplan–Meier analysis and Cox proportional hazard regression analysis. Receiver operating characteristic (ROC) curves were plotted to compare the area under the curve (AUC) of IS with other clinical parameters. Nomograms for disease-free survival (DFS) and overall survival (OS) prediction were constructed based on the identified parameters.

**Results:**

Finally, 20 (19.8%), 57 (56.4%), and 24 (23.8%) GC patients were identified with low, intermediate, and high IS levels, respectively. GC patients with higher IS levels exhibited better DFS (*p* < 0.001) and OS (*p* < 0.001). IS was an independent prognostic factor for both DFS (*p* < 0.001) and OS (*p* < 0.001) in multivariate analysis. IS presented a better predictive ability than the traditional pathological tumor-node-metastasis (pTNM) staging system (AUC: 0.801 vs. 0.677 and 0.800 vs. 0.660, respectively) with respect to both DFS and OS. The C-index of the nomograms for DFS and OS prediction was 0.737 and 0.774, respectively.

**Conclusions:**

IS is a strong predictive factor for both DFS and OS in GC patients treated with adjuvant chemoradiotherapy, which may complement the traditional pTNM staging system.

## 1. Introduction

Gastric cancer (GC) is the fifth most common malignant tumor with the third highest mortality rate worldwide [1]. Despite the innovations of modern screening methods, emerging new drugs, and multidisciplinary management, the prognosis of GC patients remains poor [2–4]. The American Joint Committee on Cancer/tumor-node-metastasis (AJCC/TNM) staging system is now widely implemented for GC treatment in clinical practice [5]. However, due to tumor heterogeneity, patients in the same TNM stage may still have different clinical outcomes. Since the prognostic information provided by the TNM staging system is insufficient, many studies have turned to other markers, such as tumor biomarkers, molecular features, or genetic signatures [6, 7]. However, only the intrinsic properties of tumors are considered in these measures, regardless of the tumor microenvironment (TME). The TME is where the host immune response occurs. Its components are quite complex and include various immune cells, fibroblasts, and tumor vasculature [8, 9]. Tumor-infiltrating lymphocytes (TILs), key components in the TME, have now been shown to be strongly correlated with patient prognosis in various cancers [10–13]. To quantify TIL infiltration, the construction of immune score (IS) was attempted. Galon et al. proposed the “Immunoscore®” system in colon cancer, which combines CD3+ and CD8+ T-cell densities from the tumor center and invasive margin [14–16]. The international validation of consensus Immunoscore® held by Pages et al. highlighted its powerful prognostic impact in patients with stage I-III colon cancer [17]. Two large cohort studies also suggested that stage III colon cancer patients with higher Immunoscore® received more benefits from chemotherapy [18, 19]. The immune score (IS) has also been investigated in lung cancer, cholangiocarcinoma, cervical cancer, gastric cancer, and other neoplasms [20–25].

Research studies on the IS for gastric cancer were attempted in several ways. Jiang et al. selected 5 (CD3_invasivemargin(IM)_, CD3_centeroftumor(CT)_, _CD_8_IM_, _CD_45ROCT, and _CD_66b_IM_) of 27 immune features using the LASSO model to build the IS system [26]. Zhang et al. combined intratumoral and stromal TILs in the scoring system [27]. Recently, Yun et al. adopted the same protocol as Pages et al. to establish the IS system in stage II/III GC patients following 5-FU-based adjuvant chemotherapy [28]. Despite the fact that the exact scoring methods used in different studies were not the same, IS based on different immune cell densities in different locations was found to have potential prognostic value in gastric cancer. However, most research studies have been restricted to GC patients who received only adjuvant chemotherapy, while patients who received adjuvant chemoradiotherapy were excluded. Whether the IS system is predictive of prognosis in these patients is still unknown.

In this study, we developed an IS system using immunohistochemistry in GC patients who received adjuvant chemoradiotherapy after curative gastrectomy, aiming to explore the prognostic significance of IS.

## 2. Materials and Methods

### 2.1. Patients

We retrospectively reviewed patients with resectable gastric cancer who underwent curative surgery followed by chemoradiation at our institute from 2006 to 2015. Criteria for inclusion were as follows: (1) histologically confirmed gastric adenocarcinoma, (2) underwent curative gastrectomy with D2 lymph node dissection, (3) received no preoperative treatment, (4) received adjuvant chemoradiotherapy, (5) adequate formalin-fixed and paraffin-embedded (FFPE) tissue blocks could be collected, and (6) complete clinical information. Patients diagnosed with metastatic lesions were excluded. Patients without adequate FFPE tissue blocks were also excluded. All these GC patients received curative D2 gastrectomy with R0 resection followed by adjuvant chemoradiation. The total dose was 45 Gy delivered in 25 fractions, 5 days per week. Patients received continuous fluorouracil infusion (225 mg/m^2^) or oral capecitabine (625 mg/m^2^) twice daily concurrently with radiotherapy from days 1 to 5 every week. Fluorouracil-based chemotherapy was performed in 1–2 cycles before and 4–6 cycles after radiotherapy. Patient clinicopathological information was retrieved, such as age, sex, Eastern Cooperative Oncology Group (ECOG) performance score, tumor location, lesion size, gastrectomy type, postoperative serum lactate dehydrogenase (LDH) level, carcinoembryonic antigen (CEA) level, histologic grade, lymphatic and vascular invasion (LVI), perineural invasion (PNI), Borrmann classification, and pT and pN staging. Borrmann classification includes type I (polypoid type), type II (ulcerated type with demarcated margin), type III (ulcerated type with peripheral infiltration), and type IV (diffuse infiltrating type) [29]. The pathologic stage was classified based on the 8th edition of the American Joint Committee on Cancer (AJCC). All patients were followed up for at least 3 years, with recurrence and death information records. Disease-free survival (DFS) was defined as the length of the period of survival without recurrence after surgery. Overall survival (OS) was calculated from the date of surgery to the date of death. This study was approved by the Ethics Committee of Fudan University Shanghai Cancer Center. The written informed consents were obtained from all patients included in this study.

### 2.2. Immunohistochemistry

For each patient, one tumor tissue block with both tumor and invasive margins was chosen and made into 4 *μ*m-thick paraffin-embedded tissue sections. Two tissue sections were dewaxed with xylene and rehydrated with decreasing concentrations of gradient alcohol. Endogenous peroxidase activity was blocked with 3% hydrogen peroxide for 15 min. Antigen retrieval was performed in the steamer for 20 min with citrate antigen retrieval solution (pH 6.0). Nonspecific antigens were blocked with 10% normal goat serum for 1 hour at 37°C. Then, the tissue sections were incubated with anti-CD3 antibody (ab5960, goat multiclonal, 1 : 200, Abcam, Cambridge, UK) and anti-CD8 antibody (#70306, mouse monoclonal, 1 : 500, Cell Signalling Technology, Danvers, MA, USA) in the wet box overnight at 4°C. Subsequently, the secondary antibody (GK5005, GTVision™ Detection System/Mo&Rb, Gene Tech, Shanghai, China) was incubated for 30 min at 37°C, and diaminobenzidine (DAB) was used for chromogen reaction. After all of the above steps, all sections were counterstained with hematoxylin-eosin solution and dehydrated with increasing xylene and alcohol gradient solutions.

### 2.3. Immune Score Construction

Finally, stained tissue sections were examined under the optical microscope. All stained sections were independently viewed by two observers who were blinded to the clinical information. We identified the tumor center (CT) and invasive margin (defined as the peritumoral area with a width of 0.6 mm, IM) under 200× magnification and chose 3 representative fields from each. The number of CD3+ or CD8+ T cells in each of the 3 fields was quantified by manual counting using ImageJ software v1.8.0 (Rawak Software Inc., Stuttgart, Germany). The cell density for each region is represented using the mean cell count of the 3 fields at 200× magnification. Then, we established the IS based on the proposal raised by Pages et al. For each case, the CD3+ and CD8+ T-cell densities were compared to the entire group and converted into percentiles. The mean of the four percentiles (two from CT and two from IM) was then translated into the immune score. Based on the three-category immune score classification, 0∼25% was classified as low, 25%∼70% was classified as intermediate, and 70∼100% was classified as high.

### 2.4. Statistical Analysis

The association between markers was analyzed using Spearman's correlation test. *r* > 0.8, 0.5 ≤ *r* < 0.8, and *r* < 0.5 were classified as high, moderate, and low correlations, respectively. The correlation between IS level and clinicopathological factors was investigated using Pearson's chi-square test or Fisher's exact test. To compare survival among groups, Kaplan–Meier curves were developed. Prognostic factors for OS and DFS were identified using univariate and multivariate Cox regression analyses. Variables identified with *p* value <0.1 in univariate analysis were included in the further multivariate analysis. The prognostic nomograms were constructed based on those identified factors, and their predictive performance was assessed by the bootstrap-corrected Harrell's concordance index (C-index) and calibration curves. To compare the predictive ability among parameters, a receiver operating characteristic (ROC) curve was generated, and the area under the curve (AUC) value was calculated. All of the above analyses were performed using IBM SPSS 25.0 (IBM Inc., Armonk, NY, USA), GraphPad Prism 9 (GraphPad Software Inc., San Diego, CA, USA), and R software v4.0.5 (R Project for Statistical Computing, Vienna, Austria). The survival package, rms package, and time ROC package were used in *R* software. All *p* values are all two-sided. A *p* value <0.05 was considered statistically significant.

## 3. Results

### 3.1. Clinicopathological Characteristics

Overall, 101 GC patients who received adjuvant chemoradiotherapy were included in our study. They had good performance status (ECOG 0–1) with 61.4% presenting ECOG 0. All of them were stage II (21.8%) or III (78.2%) according to the 7th AJCC/TNM staging system, with a median age of 55 years old (range 27–77). A minority of patients showed elevated postoperative LDH level (8.9%) or CEA level (6.9%). Most lesions (57.4%) were located in the lower part of the stomach, and the median tumor size was 4 cm (range 1–15 cm). Most patients (65.3%) underwent subtotal gastrectomy. Borrmann type III (65.3%), poor differentiation grade (66.3%), LVI positivity (62.4%), and PNI positivity (61.4%) were the most commonly observed characteristics (Supplementary Table 1). The median follow-up time was 73.7 months (range 3.6–144.4 months), and 58 relapses and 58 deaths occurred during this period.

### 3.2. TILs and Immune Score

**Figure 1:**
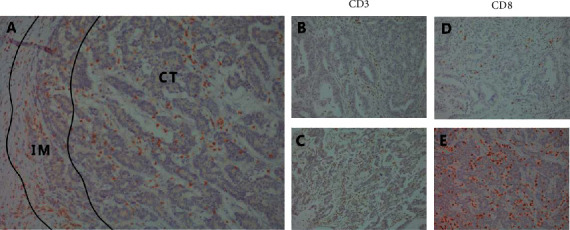
Representative images of stained CD3+ and CD8+ cells in the tumor center (CT) and invasive margin (IM) regions at 200× magnification. (a) The CT and IM regions. (b, c) Low and high CD3+ cell infiltration. (d, e) Low and high CD8+ cell infiltration.

The CD3+ and CD8+ T-cell counts in each region were quantified from stained tissue sections (representative pictures are shown in Figures 1(a)–1(e))). The median counts/20× magnification for CD3-CT, CD3-IM, CD8-CT, and CD8-IM were 123 (range 30–420), 130 (range 25–406), 110 (range 10–323), and 103 (range 10–282), respectively (Supplementary Table 1). Both CD3+ and CD8+ cell counts in the CT region were slightly higher than those in the IM region. The density of CD3+ cells in the CT region was moderately correlated with that in the IM region (*r* = 0.680, *p* < 0.001). The same results were observed in CD8+ cells (*r* = 0.681, *p* < 0.001, Supplementary Figure 1(A–D)). The immune score was constructed as previously defined based on the mean percentiles obtained. The numbers of patients classified as IS-low, IS-int, and IS-high were 20 (19.8%), 57 (56.4%), and 24 (23.8%), respectively. The association between IS and clinical parameters was explored, and patients with low IS had higher pT stage (*p* = 0.006), higher pN stage (*p* = 0.023), higher pTNM stage (*p* = 0.009), and larger lesions (*p* = 0.029) and were more likely to be LVI positive (*p* = 0.020). Although not statistically significant, patients with low IS tended to have a lower differentiation grade (*p* = 0.092) (Table 1).

### 3.3. IS and Prognosis

**Figure 2:**
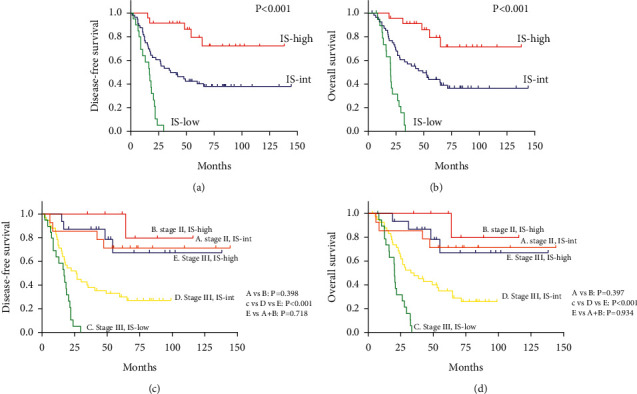
Kaplan–Meier curves grouped by IS level (low, intermediate, and high) and stage. (a) Disease-free survival (DFS) and IS level. (b) Overall survival (OS) and IS level. (c) DFS comparison stratified by IS and stage. (d) OS comparison stratified by IS and stage.

Next, the survival curves were drawnfor IS discrimination. A higher IS level indicated better DFS (p < 0.001) and OS (p < 0.001). The median DFS and median OS for IS-high group patients were not reached. The median DFS for the IS-low and IS-intermediate (IS-int) groups was 17.0 months (standard deviation, SD ± 3.1) and 40.7 months (SD ± 12.6), respectively. The median OS for the IS-low and IS-int groups was 20.4 months (standard deviation, SD ± 0.5) and 49.4 months (SD ± 9.1), respectively (Figures 2(a) and 2(b)).

Univariate and multivariate Cox regression analyses were performed to identify all prognostic factors in all GC patients included in this study. Univariate analysis revealed that CEA level (*p* = 0.008), surgery type (*p* = 0.002), lesion size (*p* = 0.076), differentiation grade (*p* = 0.014), pTNM stage (*p* = 0.001), Borrmann classification (*p* = 0.071), and IS (*p* < 0.001) were all correlated with DFS. Only pTNM stage (*p* = 0.026) and IS (*p* < 0.001) remained significant in multivariate Cox regression analysis. In OS analysis, we observed similar results. Age (*p* = 0.094), CEA level (*p* = 0.011), surgery type (*p* = 0.002), lesion size (*p* = 0.035), differentiation grade (*p* = 0.011), pTNM stage (*p* = 0.001), Borrmann classification (*p* = 0.103), PNI (*p* = 0.099), and IS (*p* < 0.001) were all correlated with OS in univariate analysis. However, only age (*p* = 0.005), pTNM (*p* = 0.029), and IS (*p* < 0.001) remained significant in multivariate analysis. These results indicate that IS is an independent prognostic factor for both DFS and OS (Tables 2 and 3).

### 3.4. Subgroup Analysis Based on Stages

When stratified by stage, IS was a strong prognostic factor in stage III GC patients (*p* < 0.001 for DFS and *p* < 0.001 for OS) but not in stage II GC patients (*p* = 0.398 for DFS and *p* = 0.397 for OS) (Figures 2(c) and 2(d)). Among all patients, stage III GC patients with low IS levels had the worst survival. Stage III GC patients with high IS levels had a similar prognosis to stage II GC patients (*p* = 0.718 for DFS; *p* = 0.934 for OS).

### 3.5. The Predictive Ability of Parameters and Prognostic Nomogram Construction

**Figure 3:**
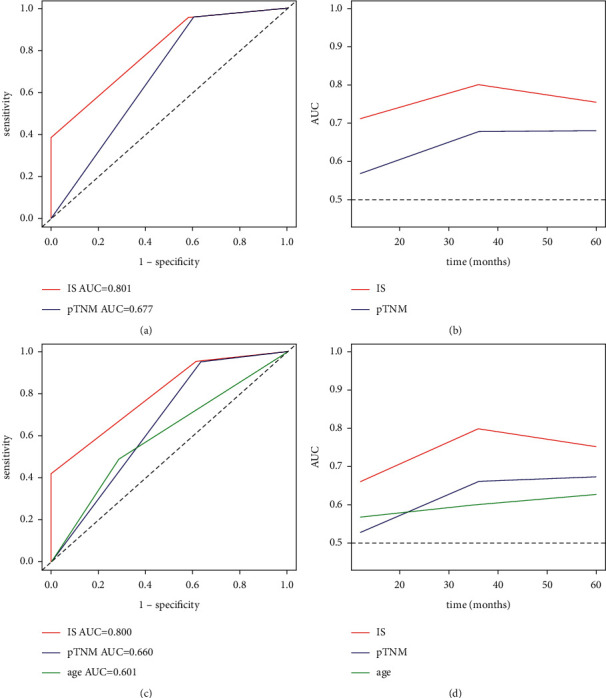
Time-dependent analysis for DFS and OS. (a) ROC curves for 3-year DFS. (b) ROC curves for 3-year OS. (c) Time-dependent ROC curves for DFS. (d) Time-dependent ROC curves for OS.

**Figure 4:**
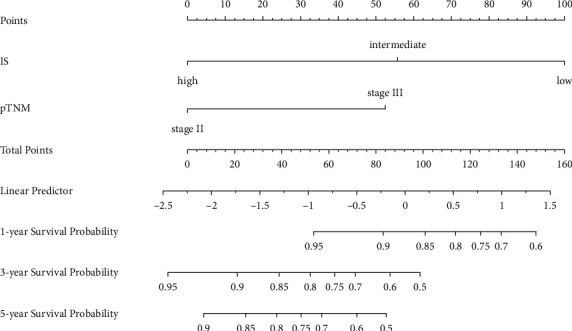
Nomogram to predict 1-year, 3-year, and 5-year DFS. Points are obtained by drawing a vertical line upward to the point axis from each variable axis including pTNM staging and IS. The sum of the points was found on the “total points” line, and a vertical line was drawn downward from here to determine the 1-, 3-, and 5-year disease progression probability. The C-index of the whole model for DFS prediction was 0.737 (95% CI, 0.729–0.745).

**Figure 5:**
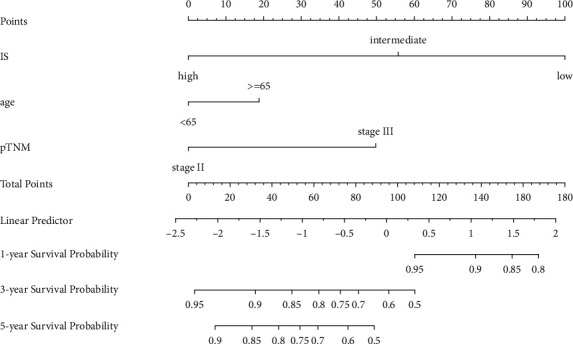
Nomogram to predict 1-year, 3-year, and 5-year OS. Points are obtained by drawing a vertical line upward to the point axis from each variable axis including age, pTNM staging, and IS. The sum of the points was found on the “total points” line, and a vertical line was drawn downward from here to determine the 1-, 3-, and 5-year survival probability. The C-index of the whole model for OS prediction was 0.774 (95% CI, 0.763–0.783).

**Figure 6:**
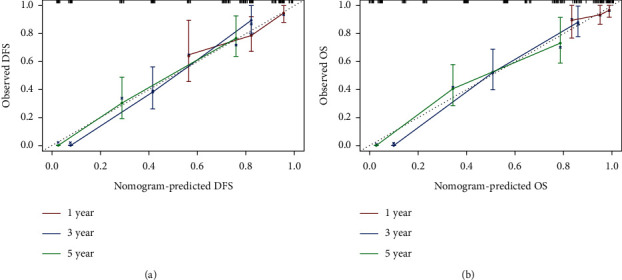
Calibration curves. (a) The calibration curve of a nomogram to predict 1-year, 3-year, and 5-year DFS. (b) The calibration curve of a nomogram to predict 1-year, 3-year, and 5-year OS.

The area under the curve (AUC) was calculated to assess the predictive ability of the identified parameters from multivariate Cox regression analysis (Figures 3(a) and 3(d)). Using 3 years as a cutoff, the predictive ability of IS (AUC 0.801 and AUC 0.800, respectively) was significantly higher than that of pTNM staging (AUC 0.677 and AUC 0.660, respectively) for both DFS and OS. The AUC was 0.601 for age in OS prediction. Though the AUC value changed with time, the predictive ability of IS was always higher than that of the pTNM staging system. The nomograms for DFS and OS prediction were constructed using the identified parameters (Figures 4 and 5). The internal validation was performed using the bootstrap method. The bootstrap-corrected C-index of the model for DFS and OS prediction was 0.737 (95% CI, 0.729–0.745) and 0.774 (95% CI, 0.763–0.783), respectively. The calibration curves showed good consistency between nomogram predicted and actually observed 1-, 3-, and 5-year DFS and OS (Figure 6).

## 4. Discussion

Gastric cancer is a highly heterogeneous cancer with a poor prognosis. Patients with the same AJCC/TNM staging can exhibit quite different clinical outcomes. Therefore, the prognostic information provided by AJCC/TNM staging is not sufficient. We need more information to guide treatment strategies in clinical practice. The tumor microenvironment, where the host immune response against tumors occurs, is composed of various components, such as immune cells, fibroblasts, and epithelial cells. Substantial studies have shown that infiltrating lymphocytes are closely correlated with patient prognosis [30, 31]. The immune score, which serves as a quantitative evaluation method for TILs, has been widely discussed. Based on the density and position of CD3+ and CD8+ cells, Galon et al. successfully established an immune scoring system in colorectal cancer [12]. Recently, the consensus Immunoscore® of colon cancer was validated in a large international, prospective, multicenter clinical trial, which showed that the IS was an independent prognostic factor for recurrence [17]. In gastric cancer, previous studies have also demonstrated the prognostic impact of immune cells, such as CD3+, CD8+, Foxp3, and CD45RO cells [32–35]. However, few studies have reported the immune score in GC, and the construction methods differed in those published essays. What is more, no study has reported an association between IS and GC patients who have received adjuvant chemoradiotherapy.

In our study, we obtained CD3+ and CD8+ cell densities from different locations using immunohistochemistry. CD3 is a specific marker for T cells, and CD8 is a specific marker for cytotoxic T cells. They were the most frequently used markers in previous studies, and research studies in gastric cancer have confirmed their role in prognosis prediction. Combining the CD3+ and CD8+ cell densities together, the TIL infiltration level is well reflected. Counts of these two markers in two different locations were obtained from stained tumor sections. A moderate correlation was found between the markers. A consensus about immune score construction has not yet been reached in gastric cancer. In some studies, a cutoff value, usually the median [22, 36], is given for each marker to distinguish low infiltrating levels (score 0) from high infiltrating levels (score 1). By combining 2 markers in 2 regions together, patients obtain scores of 0 to 4. However, the median may not be the best cut off value, and much information could be lost in this way. In this study, we established the IS system using the consensus Immunoscore® modified by Pages et al. The exact whole immune infiltration status is revealed by converted percentiles from the immune cell counts. Based on the IS levels, we classified patients into three groups, namely, IS-low (20, 19.8%), IS-intermediate (57, 56.4%), and IS-high (24, 23.8%). The number of patients in each group was relatively balanced. Through analysis, we found that lower IS was associated with higher TNM stage, larger lesions, LVI-positive state, and lower differentiation grade. It seemed that a lower IS level was associated with a heavier tumor burden, which is generally consistent with previous studies. In this study, we explored the prognostic impact of IS. Kaplan–Meier curve analysis showed that IS was predictive for DFS and OS at *p* < 0.001. IS remained an independent prognostic factor in multivariate Cox regression analysis. A higher IS level indicated better DFS and OS, which is also concordant with previous studies. In addition, the IS score was a better predictive parameter than the TNM staging system. IS can be used to complement the TNM staging system and help to identify patients who can benefit from adjuvant chemoradiotherapy. Prognostic nomograms were constructed using the identified parameters from the Cox regression analysis. Both of them had a good predictive performance with C-index >0.7, and the corresponding calibration curves showed favorable results.

Subgroup analysis was also performed. When stratified by stage, we found a prognostic impact of IS in stage III but not stage II GC patients. This result may be caused by the small sample size of only 22 stage II GC patients. Among them, there were only 14 patients with intermediate IS levels, 8 patients with high IS levels, and no patients with low IS levels. To determine the exact prognostic effect of IS, we need to include more stage II GC patients in the future. Among stage III GC patients, those with high IS levels exhibited the best survival, which was no worse than that of the stage II GC patients (*p* = 0.718 for DFS, *p* = 0.934 for OS). Although no stage II GC patients with low IS level were included here and this result still needs to be validated in larger samples, it may suggest that stage III GC patients with high IS exhibit distinct biological behaviors from others. This may help guide stratified treatment to avoid possible overtreatment.

In summary, IS was demonstrated as a strong prognostic indicator for GC patients in our study. Higher IS level, which meant higher TIL infiltration, was correlated with a better prognosis. However, how to use the immune score to guide the treatment remained unclear. Research studies on colon cancer found that those with high IS levels benefited from more cycles of adjuvant chemotherapy while those with low IS levels did not [17, 18]. This may suggest a need for more aggressive treatment in GC patients with high IS levels, which should be explored in the future. Besides, since the abscopal effect showed the great potential of radiation on the activation of the immune system, the combination of radiation and immunotherapy has been a hot issue nowadays [37, 38]. The numbers of studies about the impact of TIL infiltration on prognosis in immunotherapy-treated patients were performed [39, 40]. We should expect the potential of TIL-based IS in guiding the immunotherapy and even its combination with radiotherapy.

There are still limitations to this study. First, this was a single-center retrospective analysis with limited evidence for clinical application. Second, the relatively small sample size may lead to bias. Third, the stained cells were counted manually, and this human error cannot be overlooked. Fourth, we may try to include more immune markers such as CD45RO, Foxp3, and PD-L1 to improve the IS. In addition, gene signatures like HER-2 and MSS state may also be included to explore its correlation with the tumor microenvironment. A multicenter prospectively designed study is required to further confirm the feasibility and reproducibility of these findings.

## 5. Conclusions

We established an immune scoring system in GC patients who received post-surgical chemoradiation in our study. Patients with high immune scores were more likely to have lower pT stages, lower pN stages, smaller lesions, and LVI-negative states. The immune score was found to be a powerful prognostic factor for both DFS and OS, which may complement the TNM system to better identify GC patients who may benefit from adjuvant chemoradiotherapy.

## Figures and Tables

**Table 1:** Correlation between IS and clinical parameters.

Variables	Immunoscore			*p* value
	Low	Median	High	
	*n* = 20	*n* = 57	*n* = 24	
	(19.8%)	(56.4%)	(23.8%)	
*Sex*				
Male	15(21.4%)	39(55.7%)	16(22.9%)	0.876
Female	5(16.1%)	18(58.1%)	8(25.8%)	

*Age*				0.311
<65	11(17.7%)	33(53.2%)	18(29.0%)	
≥65	9(23.1%)	24(61.5%)	6(15.4%)	

*ECOG*				0.433
0	13(21.0%)	32(51.6%)	17(27.4%)	
1	7(17.9%)	25(64.1%)	7(17.9%)	

*LDH (U/L)*				0.999
<250	18(20.2%)	50(56.2%)	21(23.6%)	
≥250	2(22.2%)	5(55.6%)	2(22.2%)	
Unknown	0(0.0%)	2(66.7%)	1(33.3%)	

*CEA (ng/ml)*				0.492
<5	16(19.3%)	45(54.2%)	22(26.5%)	
≥5	1(14.3%)	6(85.7%)	0(0.0%)	
Unknown	3(30.0%)	5(50.0%)	2(20.0%)	

*Tumor location*				0.248
Upper 1/3	5(23.8%)	9(42.9%)	7(33.3%)	
Middle 1/3	4(26.7%)	10(66.7%)	1(6.7%)	
Lower 1/3	9(15.5%)	33(56.9%)	16(27.6%)	
Total stomach	2(28.6%)	5(71.4%)	0(0.0%)	

*Tumor size (cm)*				**0.029**
<5	7(12.3%)	32(56.1%)	18(31.6%)	
≥5	13(29.5%)	25(56.8%)	6(13.6%)	

*Surgery type*				**0.010**
Subtotal gastrectomy	8(12.1%)	38(56.7%)	20(30.3%)	
Total gastrectomy	12(34.3%)	19(54.3%)	4(11.4%)	

*Differentiation grade*				0.092
Moderate	3(8.8%)	20(58.8%)	11(32.4%)	
Poor	17(25.4%)	37(55.2%)	13(19.4%)	

*Borrmann classification*				0.339
I	0(0%)	2(66.7%)	1(33.3%)	
II	3(15.8%)	9(47.4%)	7(36.8%)	
III	14(21.2%)	36(54.5%)	16(24.2%)	
IV	3(23.1%)	10(76.9%)	0(0%)	

*pT*				**0.006**
1	0(0.0%)	1(16.7%)	5(83.3%)	
2	0(0.0%)	6(66.7%)	3(33.3%)	
3	4(11.8%)	23(67.6%)	7(20.6%)	
4	16(30.8%)	27(51.9%)	9(17.3%)	

*pN*				**0.023**
0	0(0.0%)	5(71.4%)	2(28.6%)	
1	0(0.0%)	10(90.9%)	1(9.1%)	
2	4(15.4%)	11(42.3%)	11(42.3%)	
3	16(28.1%)	13(54.4%)	10(17.5%)	

*pTNM*				**0.009**
Stage II	0(0.0%)	14(63.6%)	8(36.4%)	
Stage III	20(25.3%)	43(54.4%)	16(20.3%)	

*LVI*				**0.020**
Negative	2(5.4%)	24(64.9%)	11(29.8%)	
Positive	18(28.1%)	33(51.6%)	13(20.3%)	

*PNI*				0.673
Negative	6(15.4%)	23(59.0%)	10(25.6%)	
Positive	14(22.6%)	34(54.8%)	14(22.6%)	

Values in bold with *p* < 0.05. LVI, lymphatic and vascular invasion; PNI, perineural invasion.

**Table 2:** Univariate and multivariate Cox regression analysis for disease-free survival.

Variables	Univariate analysis		Multivariate analysis	
	HR (95% CI)	*p* value	HR (95% CI)	*p* value
Sex (F/M)	0.796 (0.452, 1.402)	0.430	—	—
Age (≥65/<65)	1.641 (0.868, 3.099)	0.127	—	—
ECOG (1/0)	1.221 (0724, 2.060)	0.454	—	—
LDH(≥250/<250 U/L)	0.864 (0.311, 2.395)	0.778	—	—
CEA(≥5/<5 ng/ml)	3.002 (1.331, 6.863)	**0.008**	2.491 (0.942, 6.585)	0.066
Tumor location		0.266	—	—
Upper 1/3	reference			
Middle 1/3	1.581 (0.671, 3.729)	0.295		
Lower 1/3	0.928 (0.467, 1.843)	0.831		
Total stomach	1.953 (0.676, 5.643)	0.216		
Surgery type (total/subtotal)	2.332 (1.378, 3.946)	**0.002**	1.429 (0.760, 2.690)	0.268
Size (≥5/<5 cm)	1.597 (0.953, 2.676)	0.076	0.835 (0.468, 1.489)	0.542
Grade (poor/moderate)	2.1 (1.147, 3.846)	**0.014**	1.055(0.505, 2.203)	0.886
Borrmann classification		0.071		0.151
I	<0.001	0.977	<0.001	0.977
II	0.278 (0.108, 0.719)	0.008	0.212 (0.055, 0.819)	0.024
III	0.567 (0.291, 1.107)	0.096	0.548 (0.263, 1.140)	0.108
IV	reference		reference	
pTNM(III/II)	4.720 (1.879, 11.859)	**0.001**	3.381 (1.156, 9.891)	**0.026**
LVI (+/−)	1.266 (0.731, 2.194)	0.399		—
PNI (+/−)	1.490 (0.860, 2.582)	0.155		—
IS		**<0.001**		**<0.001**
Low	reference		reference	
Intermediate	0.269 (0.144, 0.500)	<0.001	0.338 (0.163,0.703)	0.004
High	0.067 (0.024, 0.188)	<0.001	0.124 (0.041, 0.375)	<0.001

Values in bold indicate *p* < 0.05. CI, confidence interval; HR, hazard ratio; F/M, female/male; LVI, lymphatic and vascular invasion; PNI, perineural invasion.

**Table 3:** Univariate and multivariate Cox regression analysis for overall survival.

Variables	Univariate analysis		Multivariate analysis	
	HR (95% CI)	*p* value	HR (95% CI)	*p* value
Sex (F/M)	0.819 (0.465, 1.442)	0.489	—	—
Age (≥65/<65)	1.723 (0.912, 3.256)	0.094	3.317 (1.430, 7.694)	**0.005**
ECOG (1/0)	1.169 (0.693, 1.972)	0.559	—	—
LDH (≥250/<250 U/L)	0.816 (0.293, 2.271)	0.697	—	—
CEA (≥5/<5 ng/ml)	2.881 (1.273, 6.523)	**0.011**	2.107 (0.798, 5.563)	0.132
Tumor location		0.189	—	—
Upper 1/3	reference			
Middle 1/3	1.748 (0.741, 4.121)	0.202		
Lower 1/3	0.966 (0.487, 1.917)	0.922		
Total stomach	2.114 (0.733, 6.096)	0.166		
Surgery type (total/subtotal)	2.287 (1.354, 3.864)	**0.002**	1.738 (0.871, 3.468)	0.117
Size (≥5/<5 cm)	1.103 (1.007, 1.207)	**0.035**	0.605 (0.325, 1.129)	0.114
Grade (poor/moderate)	2.187 (1.192, 4.010)	**0.011**	1.457 (0.646, 3.286)	0.364
Borrmann classification		0.103		0.266
I	<0.001	0.973	<0.001	0.977
II	0.301 (0.116,0.779)	0.013	0.284 (0.074, 1.092)	0.067
III	0.586 (0.300,1.145)	0.118	0.537 (0.252, 1.144)	0.107
IV	reference		reference	
pTNM (III/II)	4.685 (1.866, 11.762)	**0.001**	3.407 (1.135, 10.228)	**0.029**
LVI (+/−)	1.278 (0.738, 2.214)	0.381	—	—
PNI (+/−)	1.588 (0.916, 2.754)	0.099	0.955 (0.504, 1.965)	0.988
IS		**<0.001**		**<0.001**
Low	reference		reference	
Intermediate	0.226 (0.122, 0.417)	<0.001	0.256 (0.119, 0.549)	<0.001
High	0.068 (0.024, 0.189)	<0.001	0.087 (0.028, 0.273)	<0.001

Values in bold with *p* < 0.05. CI, confidence interval; HR, hazard ratio; F/M, female/male; LVI, lymphatic and vascular invasion; PNI, perineural invasion.

## Data Availability

The data used to support the findings of this study are included within the article.

## Supplementary Materials

Supplementary Materials Supplementary Table 1: baseline clinicopathological characteristics of the patients. Supplementary Figure 1: correlation among immune cells. The *r* ratio and *p* value were calculated using Spearman's correlation test. (A) CD3-CT and CD3-IM. (B) CD8-CT and CD8-IM. (C) CD3-CT and CD8-CT. (D) CD3-IM and CD8-IM.
