# Supplementary material for: Immune Score Predicts Outcomes of Gastric Cancer Patients Treated with Adjuvant Chemoradiotherapy
Source: J Oncol. 2021 Dec 27;2021:9344124. doi: 10.1155/2021/9344124 (PMC8723845; doi:10.1155/2021/9344124)
Supplement: Supplementary Materials — Supplementary Table 1: baseline clinicopathological characteristics of the patients. Supplementary Figure 1: correlation among immune cells. The r ratio and p value were calculated using Spearman's correlation test. (A) CD3-CT and CD3-IM. (B) CD8-CT and CD8-IM. (C) CD3-CT and CD8-CT. (D) CD3-IM and CD8-IM. [file 9344124.f1.docx]

Supplementary Table 1: Baseline clinicopathological characteristics of the patients.

| Variables | N |
| --- | --- |
| Sex  Male  Female  Age (y)  <60  ≥60  ECOG  0  1  LDH (U/L)  <250  ≥250  unknown  CEA (ng/ml)  <5  ≥5  unknown  Tumor location  Upper 1/3  Middle 1/3  Lower 1/3  Total stomach  Operation type  Subtotal gastrectomy  Total gastrectomy  Tumor size (cm)  <5  ≥5  Differentiation grade  moderate  poor  Borrmann Classification  I  II  III  IV  pT  1  2  3  4  pN  0  1  2  3  pTNM  II  III  LVI  Negative  Positive  PNI  Negative  Positive  TILs (median, range/20× magnification)  CD3-CT  CD3-IM  CD8-CT  CD8-IM | 70(69.3%)  31(60.7%)  62(61.4%)  39(38.6%)  62(61.4%)  39(38.6%)  89(88.1%)  9(8.9%)  3(3.0%)  83(82.2%)  7(6.9%)  11(10.9%)  21(20.8%)  15(14.9%)  58(57.4%)  7(6.9%)  66(65.3%)  35(34.7%)  57(56.4%)  44(43.6%)  34(33.7%)  67(66.3%)  3(3.0%)  19(18.8%)  66(65.3%)  13(12.9%)  6(6.0%)  9(8.9%)  34(33.7%)  52(51.5%)  7(6.9%)  11(10.9%)  26(25.7%)  57(56.4%)  22(21.8%)  79(78.2%)  37(36.6%)  63(62.4%)  39(38.6%)  62(61.4%)  123 (range 30-420)  130 (range 25-406)  110 (range 10-323)  103 (range 10-282) |

LVI: lymphatic and vascular invasion, PNI: Perineural invasion, TILs: tumor infiltrating lymphocytes, CT: tumor center, IM: invasive margin.


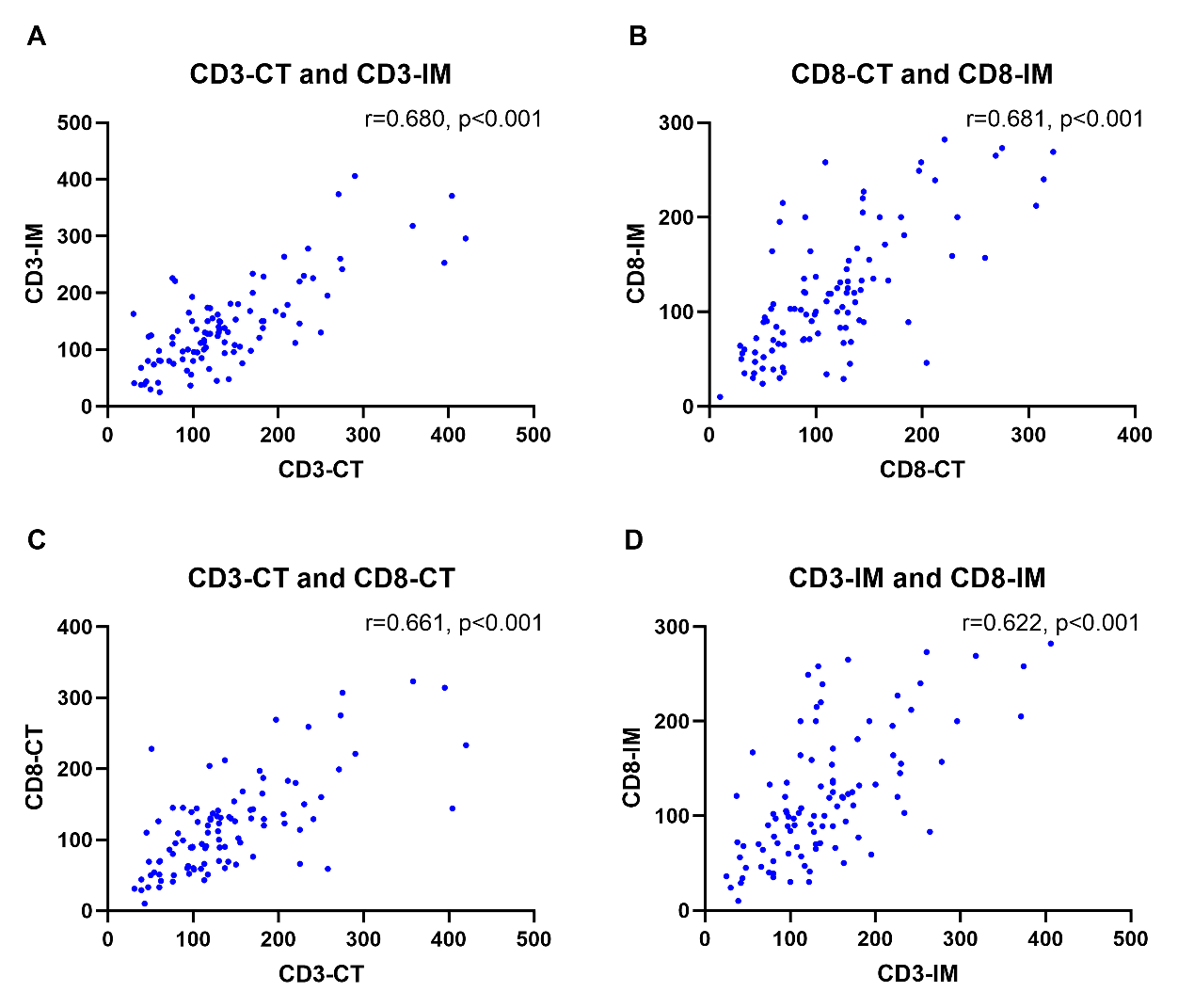


Supplementary Figure 1: Correlation among immune cells. The r ratio and P-value were calculated using Spearman’s correlation test. (A) CD3-CT and CD3-IM. (B) CD8-CT and CD8-IM. (C) CD3-CT and CD8-CT. (D) CD3-IM and CD8-IM.
